# Automatic craniomaxillofacial landmarks detection in CT images of individuals with dentomaxillofacial deformities by a two-stage deep learning model

**DOI:** 10.1186/s12903-023-03446-5

**Published:** 2023-11-17

**Authors:** Leran Tao, Meng Li, Xu Zhang, Mengjia Cheng, Yang Yang, Yijiao Fu, Rongbin Zhang, Dahong Qian, Hongbo Yu

**Affiliations:** 1 Department of Oral and Cranio-maxillofacial Surgery, Shanghai Ninth People’s Hospital, College of Stomatology, Shanghai Jiao Tong University School of Medicine, Shanghai, 200011 China; 2 National Center for Stomatology & National Clinical Research Center for Oral Diseases, Shanghai, 200011 China; 3 Shanghai Key Laboratory of Stomatology & Shanghai Research Institute of Stomatology, Shanghai, 200011 China; 4 Mechanical college, Shanghai Dianji University, Shanghai, 201306 China; 5 College of Stomatology, Shanghai Jiao Tong University School of Medicine, Shanghai, 200125 China; 6 Shanghai Lanhui Medical Technology Co., Ltd, Shanghai, 200333 China; 7 School of Biomedical Engineering, Shanghai Jiao Tong University, Shanghai, 200030 China

**Keywords:** Dentomaxillofacial deformity, Cephalometric analysis, Deep learning, Landmarks detection, Computer-assisted surgery design.

## Abstract

**Background:**

Accurate cephalometric analysis plays a vital role in the diagnosis and subsequent surgical planning in orthognathic and orthodontics treatment. However, manual digitization of anatomical landmarks in computed tomography (CT) is subject to limitations such as low accuracy, poor repeatability and excessive time consumption. Furthermore, the detection of landmarks has more difficulties on individuals with dentomaxillofacial deformities than normal individuals. Therefore, this study aims to develop a deep learning model to automatically detect landmarks in CT images of patients with dentomaxillofacial deformities.

**Methods:**

Craniomaxillofacial (CMF) CT data of 80 patients with dentomaxillofacial deformities were collected for model development. 77 anatomical landmarks digitized by experienced CMF surgeons in each CT image were set as the ground truth. 3D UX-Net, the cutting-edge medical image segmentation network, was adopted as the backbone of model architecture. Moreover, a new region division pattern for CMF structures was designed as a training strategy to optimize the utilization of computational resources and image resolution. To evaluate the performance of this model, several experiments were conducted to make comparison between the model and manual digitization approach.

**Results:**

The training set and the validation set included 58 and 22 samples respectively. The developed model can accurately detect 77 landmarks on bone, soft tissue and teeth with a mean error of 1.81 ± 0.89 mm. Removal of region division before training significantly increased the error of prediction (2.34 ± 1.01 mm). In terms of manual digitization, the inter-observer and intra-observer variations were 1.27 ± 0.70 mm and 1.01 ± 0.74 mm respectively. In all divided regions except Teeth Region (TR), our model demonstrated equivalent performance to experienced CMF surgeons in landmarks detection (*p*  >  0.05).

**Conclusions:**

The developed model demonstrated excellent performance in detecting craniomaxillofacial landmarks when considering manual digitization work of expertise as benchmark. It is also verified that the region division pattern designed in this study remarkably improved the detection accuracy.

**Supplementary Information:**

The online version contains supplementary material available at 10.1186/s12903-023-03446-5.

## Background

For the reason of abnormal jaw development in terms of size, shape and the positional relationship between maxilla and mandible, patients with dentomaxillofacial deformities suffer from malocclusion, facial abnormality related dysfunctions, and etc. The combination of orthognathic and orthodontics treatment can rehabilitate occlusal function and harmony facial profile. Given the complexity and diversity of dentomaxillofacial deformities, accurate diagnosis and precise surgical planning are indispensable.

Cephalometric analysis is commonly used in the diagnosis and surgical planning. The cephalometric analysis of CT images provides more information than lateral cephalogram (X-ray image) because CT images can be reconstructed into a 3-Dimensional (3D) skull model [1]. However, the application of 3D cephalometric analysis is limited in clinic for the reason that it is time-consuming and labour-intensive.

Deep learning (DL) provides us new solutions to these challenges and has already demonstrated its great potential in 2-Dimensional (2D) cephalometric analysis based on X-ray images [2–5]. In recent years, research on automatic 3D cephalometric analysis based on CT or cone beam CT (CBCT) has been more and more popular. Due to the high graphic memory footprint to process 3D images and the clinical need for high detection accuracy, dividing original images into multiple sub-regions is a promising strategy. In research of G. Dot et al. (2022), input image resolution was preserved in their proposed model by defining 5 regions of interest (ROI) as coarsely predicted localization of the landmarks. However, their model involved few tooth landmarks and did not include any landmarks in facial soft tissue, which are indispensable for cephalometric analysis [6]. Lang et al. (2022) came up with a three-stage coarse-to-fine framework and managed to reduce the prediction error to 1.38 ± 0.95 mm. However, it inevitably increased the operating time [7]. Employing lightweight networks like 3D U-Net [8] or V-Net [9] is one way to reduce graphics memory footprint. Liu et al. (2021) employed 3D U-Net for landmarks detection, while the 3D U-Net also could not process the origin image. They had to decrease the resolution of CT images to 96 × 96 × 96 to maintain training process, and finely tune the detection result in another detection stage [10].

Driven by clinical demands and limitations of previous researches, this study aimed to develop a two-stage landmarks detection model to accomplish automatic and accurate 3D cephalometric analysis under low graphic memory footprint, especially targeting patients with dentomaxillofacial deformities. To promise clinical practicability of the method, we included 77 landmarks on bone, teeth and facial soft tissue in the detection task. To minimize graphics memory footprint while optimizing prediction accuracy, a recently proposed lightweight network named 3D UX-Net [11] was employed and a new region division pattern was designed in this model.

## Materials and methods

### Data preparation

In this study, 80 sets of CT data of patients with dentomaxillofacial deformities were selected from Shanghai Ninth People’s Hospital (Shanghai, China). This research was approved by the Research Ethics Committee of Hospital (IRB No. SH9H-2022-TK12-1). The inclusion criteria were: (1) patients diagnosed with dentomaxillofacial deformity and orthognathic-orthodontic joint treatment were required; (2) CT scanned before treatment. The exclusion criteria were: (1) congenital dentofacial deformities; (2) have a history of orthognathic treatment. Each CT had a pixel size of 0.45 mm x 0.45 mm, a slice interval of 1 mm, and a resolution of 512 × 512 × 231. To reduce graphics memory footprint in computational process, CT images were resampled and the pixel was resized to 1 mm x 1 mm, so that each CT had a resolution of 229 × 229 × 231.

**Fig. 1:**
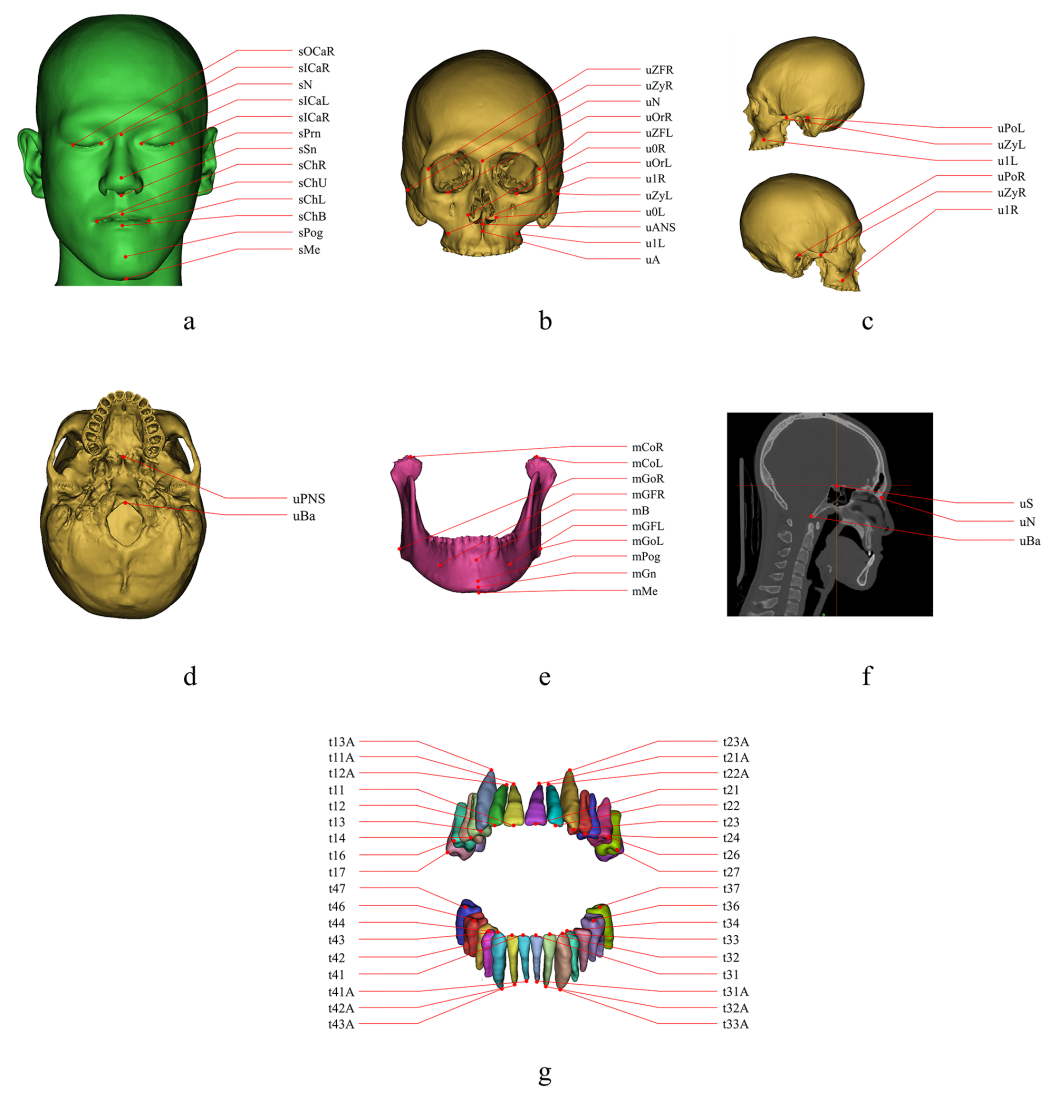
Name and locations of 77 CMF landmarks. **a.** 13 facial soft tissue landmarks. **b-f.** 28 skeletal landmarks. **g.** 36 dental landmarks

Based on clinical requirements and researches involved 3D cephalometric analysis [12–14], 77 landmarks, including 13 facial soft tissue landmarks, 28 skeletal landmarks and 36 dental landmarks, were included in the detection task. The names, defintions and locations of the selected CMF landmarks were shown in detail (Fig. 1, Supplementary Table 1). 77 landmarks were manually digitized in all CT images by 2 junior CMF surgeons and modified by a senior CMF surgeon using Mimics software (Materialise, Belgium). Then, the final labelling results were exported in xml format as the ground truth for model training.

### Model architecture

**Fig. 2:**
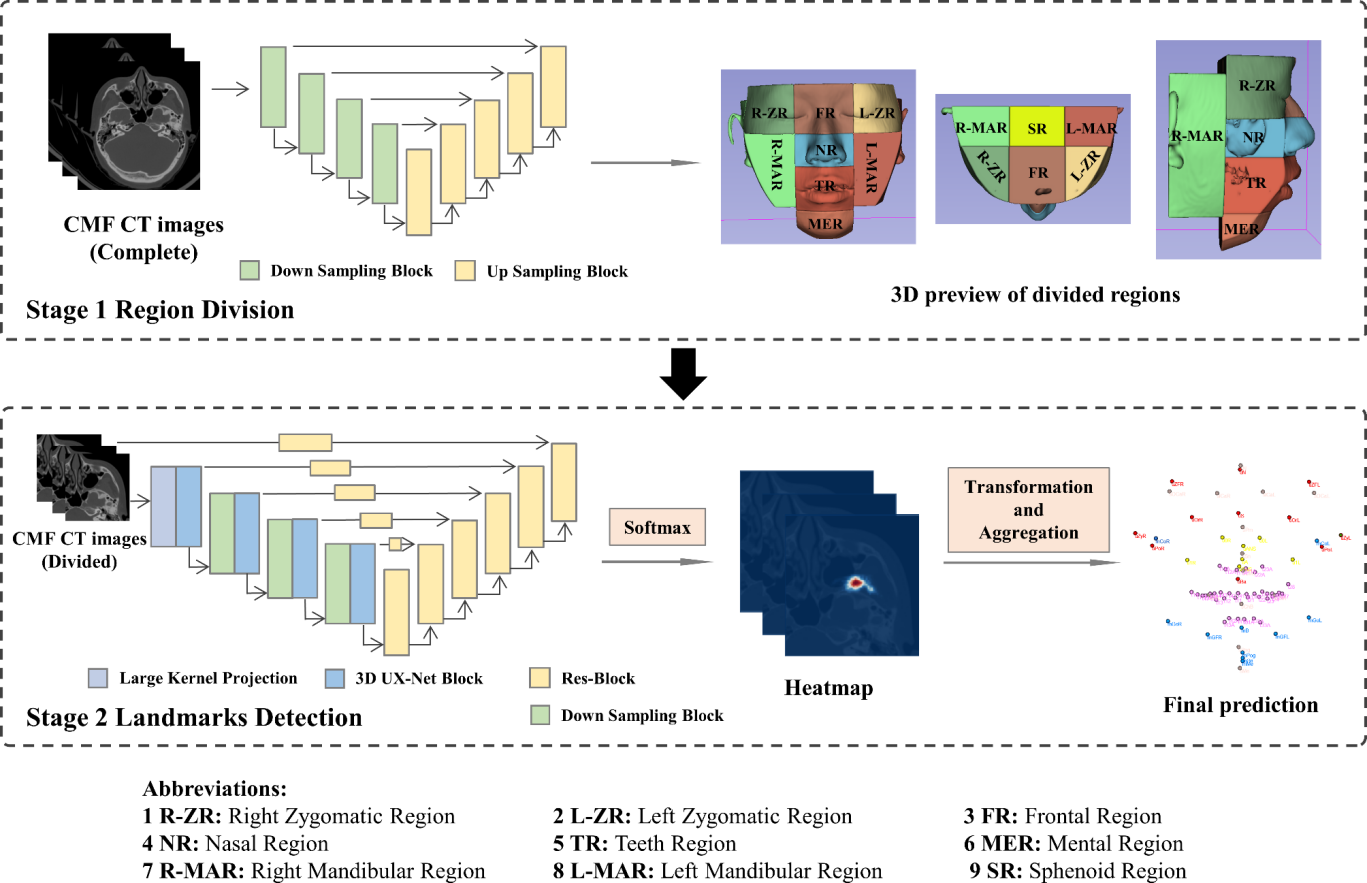
Overview of the proposed two-stage model for detecting 77 landmarks from CT images. The first stage is to divide original CT images into 9 regions using V-Net. The second stage is to detect landmarks using 3D UX-Net.

A two-stage deep learning model was proposed (Fig. 2). In Stage 1, a region division neural network was utilized to divide original CT images into 9 sub-regions based on the region division pattern we designed. In Stage 2, landmarks detection neural networks were constructed to propose possible location of each landmark based on sub-regions obtained in Stage 1.

### Stage 1: region division neural network

A new region division pattern was designed based on the feature of craniomaxillofacial structures. Compared to the ROI detection pattern in [6], it divided the skull into adjacent sub-regions to adapt for more and scattered landmarks detection. To create the annotations for the region division, location of some representative landmarks which were pre-annotated to segment the image were used, as shown in the Supplementary Fig. 1. By employing a classic segmentation neural network, V-Net, the skull was divided into 9 anatomical regions, as shown in Fig. 2 (Stage 1). Instead of directly compressing the image to reduce graphic memory footprint, adopting region division pattern can preserve original image resolution. Therefore, more graphic information was able to be identified by the landmarks detection neural network, contributing to more accurate landmarks detection results.

### Stage 2: landmarks detection neural network

The landmarks detection network (Stage 2) employed 3D UX-Net as the backbone. The basic network architecture of 3D UX-Net consists of large kernel projection layer, encoder and decoder section. At the same time, skip connections are set to avoid information loss and gradient disappearance. Input data were divided into small patch data by large kernel projection layer, and patch-wise features were extracted as the input of the encoder section. The encoder section contains four 3D UX-Net blocks and four downsampling blocks. Large convolutional kernels (7 × 7 × 7) and small convolutional kernels (1 × 1 × 1) were included in 3D UX-Net blocks to enlarge global receptive field and supplement additional contextual information. Sixteen-fold image compression was implemented by four downsampling blocks to reduce the computational volume and reserve sufficient semantic feature. The decoder section was set to recover image resolution through res-blocks and long skip connections. After applying Softmax function, landmark heatmaps were obtained and the voxel with the highest probability value in each landmark heatmap was proposed as final predicted landmark. The architecture and components of the model, as well as the input and output dimensions of each layer, was illustrated in Supplementary Fig. 2. Since the input and output dimensions vary for different regions, frontal region (FR) was used as an example.

### Implementation details

The training set and validation set included 58 and 22 samples respectively. Abnormal data (reasons for the abnormal outcomes will be analysed in the Discussion section) were eliminated using the median absolute deviation (MAD) algorithm. The model was validated in validation set every 10 epoch and mean error of the validation set was taken as the result. Input and output size of different regions were included in Supplementary Table 2. The training details of Stage 1 neural network were set as follows: Optimizer: Adam, learning rate: 0.003, loss: Dice focal loss, batch size: 2, epochs: 300. The training details of Stage 2 neural network were set as follows: Optimizer: Adam, learning rate: 0.0001, loss: focal loss, batch size: 2, epochs: 500.

### Performance evaluation

The evaluation metric we chose for prediction performance was prediction error, which was Euclidean distance between the coordinates of predicted landmarks and ground truth. Prediction error was calculated using Eq. 1:

1 $${l_e} = \frac{1}{n}\sum\limits_{i = 1}^n {{{\left[ {{{(x_{pre}^i - x_{gt}^i)}^2} + {{(y_{pre}^i - y_{gt}^i)}^2} + {{(z_{pre}^i - z_{gt}^i)}^2}} \right]}^{\frac{1}{2}}}}$$

Where l_e_ represents the prediction error, (x_pre_, y_pre_, z_pre_) represents the predicted coordinates, (x_gt_, y_gt_, z_gt_) represents the ground truth, and n represents the number of validation samples.

To compare the landmarks detection performance of 3D UX-Net and V-Net, model training process was also implemented with the backbone of Stage 2 neural network substituted with V-Net. Furthermore, an experiment was conducted to evaluate the impact of region division on the final predicted results. In order to evaluate the effectiveness of our proposed model in clinical practice, we asked two CMF surgeons to manually digitize all 77 landmarks on 10 CT images from training set, and one of them to repeat the work on the same 10 CT images one week later. Inter-observer and intra-observer variations were calculated based on the results.

## Results

### Prediction performance in four different settings

#### Setting 1: v-net without region division

Without region division and at a resolution of 96 × 96 × 96, the model with V-Net as the backbone in Stage 2 had a mean error of 2.40 ± 1.08 mm (Table 1), and 22.08% of the 77 landmarks fell within 2 mm, 59.74% within 2.5 mm, 87.01% within 3 mm, and 96.10% within 4 mm (Table 2).

#### Setting 2: 3D UX-net without region division

Without region division and at a resolution of 96 × 96 × 96, the model with 3D UX-Net as the backbone in Stage 2 had a mean error of 2.34 ± 1.01 mm (Table 1), and 35.06% of the 77 landmarks fell within 2 mm, 61.04% within 2.5 mm, 85.71% within 3 mm, and 96.10% within 4 mm (Table 2).

#### Setting 3: v-net with region division

With region division and at a resolution of 229 × 229 × 231, the model with V-Net as the backbone in Stage 2 had a mean error of 1.90 ± 0.93 mm (Table 1), and 61.04% of the 77 landmarks fell within 2 mm, 89.61% within 2.5 mm, 96.10% within 3 mm, and 98.70% within 4 mm (Table 2). This setting had a similar graphic memory footprint to Setting 1.

#### Setting 4: 3D UX-net with region division

With region division and at a resolution of 229 × 229 × 231, the model with 3D UX-Net as the backbone in Stage 2 had a mean error of 1.81 ± 0.89 mm (Table 1), and 76.62% of the 77 landmarks fell within 2 mm, 90.91% within 2.5 mm, 93.51% within 3 mm, and 98.70% within 4 mm (Table 2). This setting had a similar graphic memory footprint to Setting 2 and demonstrated the best performance for landmarks detection.

**Fig. 3:**
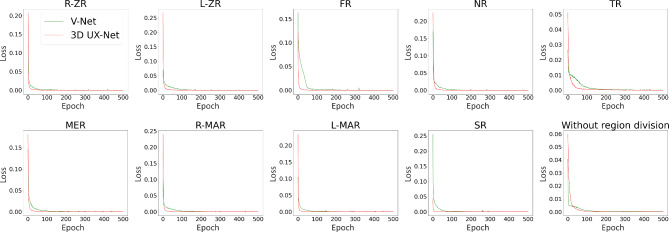
Training loss curve in 4 different settings

**Fig. 4:**
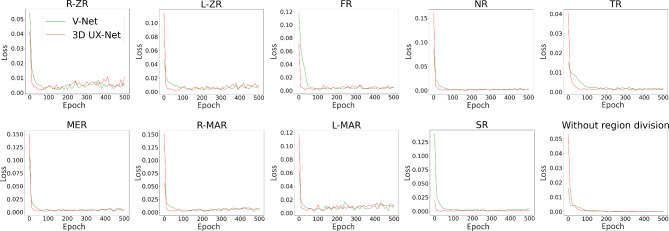
Validation loss curve in 4 different settings

**Fig. 5:**
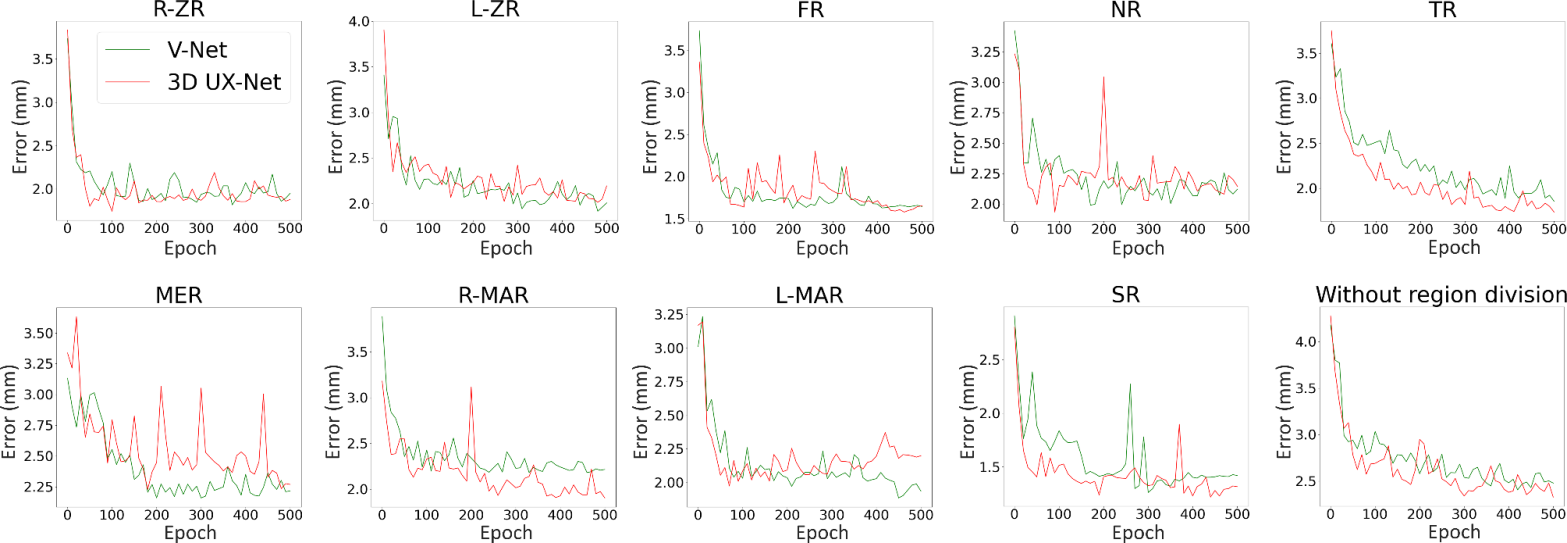
Validation error curve in 4 different settings

The training loss curve (Fig. 3), validation loss curve (Fig. 4) and validation error curve (Fig. 5) were demonstrated as follows.

**Table 1:** Prediction errors in 4 different settings (unit: mm)

	R-ZR	L-ZR	FR	NR	TR	MER	R-MAR	L-MAR	SR	Overall
**V-Net without** **Region division**										2.40 ± 1.08
**3D UX-Net without** **Region division**										2.34 ± 1.01
**V-Net with** **Region division**	1.82 ± 0.84	1.92 ± 0.93	1.61 ± 0.80	1.99 ± 1.08	1.89 ± 0.94	2.16 ± 1.01	2.18 ± 0.99	1.89 ± 0.84	1.26 ± 0.52	1.90 ± 0.93
**3D UX-Net with** **Region division**	1.74 ± 0.82	2.02 ± 0.98	1.58 ± 0.77	1.93 ± 0.96	1.74 ± 0.88	2.23 ± 1.09	1.90 ± 0.96	1.97 ± 0.89	1.22 ± 0.44	**1.81** **± 0.89**

**Table 2:** Prediction accuracy within a given margin

	Error margin	Prediction accuracy
**V-Net without** **Region division**	2 mm	22.08%
	2.5 mm	59.74%
	3 mm	87.01%
	4 mm	96.10%
**3D UX-Net without** **Region division**	2 mm	35.06%
	2.5 mm	61.04%
	3 mm	85.71%
	4 mm	96.10%
**V-Net with** **Region division**	2 mm	61.04%
	2.5 mm	89.61%
	3 mm	96.10%
	4 mm	98.70%
**3D UX-Net with** **Region division**	2 mm	**76.62%**
	2.5 mm	90.91%
	3 mm	93.51%
	4 mm	98.70%

### Comparison with manually digitized landmarks

The error of landmarks detection using 3D UX-Net with region division was compared to the inter- or intra-observer variation. The inter-observer and intra-observer variations were 1.27 ± 0.70 mm and 1.01 ± 0.74 mm, respectively. The intraclass correlation coefficient of landmarks digitized by the two observers was greater than 0.99. Unpaired t-tests proved that there is no statistically significant difference between prediction error of the model and inter-observer variation except for teeth region (TR) (*p* > 0.05) (Table 3). Using the form of scatter plots, differences in each of the nine regions are shown in Fig. 3. In TR, errors of the model and inter-observer variation were 1.73 ± 0.84 mm and 0.71 ± 0.53 mm, which showed a statistically significant difference (*p* < 0.05).

**Table 3:** Comparison between prediction error of model and inter-observer variation (unit: mm)

Region	Prediction error of the model		Inter-observer variation		Mean difference	*p*-value
	Mean	Std	Mean	Std		
**R-ZR**	1.74	0.82	2.09	1.23	-0.35	＞0.05
** L-ZR**	2.02	0.98	1.98	0.87	0.04	＞0.05
**FR**	1.58	0.77	1.50	0.90	0.08	＞0.05
**NR**	1.93	0.96	1.69	0.78	0.24	＞0.05
**TR**	1.74	0.88	0.71	0.53	1.03	<0.05
**MER**	2.23	1.09	2.26	0.94	-0.03	＞0.05
**R-MAR**	1.90	0.96	1.99	0.87	-0.09	＞0.05
** L-MAR**	1.97	0.89	2.28	0.86	-0.31	＞0.05
**SR**	1.22	0.44	0.85	0.61	0.37	＞0.05

**Fig. 6:**
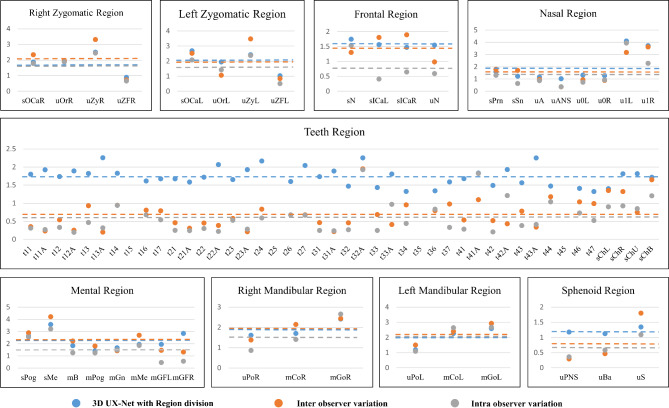
Differences of prediction error among the model, inter-observer variation and intra-observer variation. (The height of the points in the scatter plot represented the average error of the landmarks detection, and the dashed line represented the average error of all the landmarks in the region)

### Precision analysis for cephalometric indicators

To better explore the applicability of the model in clinic, a precision analysis was conducted for 6 indicators (3 angle indicators and 3 distance indicators) commonly used in cephalometric analysis, including SNB, SNA, ANB, and three sides of the mGoR-uN-mGoL triangle. The values of these indicators were calculated by the coordinates of related landmarks, which were obtained by the model prediction or manual annotation (i.e. ground truth). 22 samples in validation set were used in analysis. Paired t-tests showed that no statistically significant difference between the predictions of this model and the ground truth in all six cephalometric indicators (Table 4).

**Table 4:** Error of indicators used in cephalometric analysis

Indicators	Model		Ground Truth		Mean difference	*p*-value
	Mean	Std	Mean	Std		
**SNA (°)**	81.96	3.24	81.64	3.33	0.32	＞0.05
**SNB (°)**	83.79	5.25	83.18	4.75	0.61	＞0.05
**ANB (°)**	3.82	1.99	3.91	1.97	-0.09	＞0.05
**mGoL-uN (mm)**	118.57	8.63	118.28	8.77	0.29	＞0.05
**mGoR-uN (mm)**	118.14	8.17	118.79	7.70	-0.65	＞0.05
**mGoL-mGoR (mm)**	96.33	4.63	96.91	5.18	-0.58	＞0.05

## Discussion

With region division and 3D UX-Net, the performance of the proposed model in landmarks digitization was equivalent to that of experienced CMF surgeons, except for TR. In TR, landmarks were manually digitized based on highly precise optical dental models that were registered to CT images, while such optical dental models could not be processed by this model. Moreover, the landmarks on the teeth are easier to be recognized than in other anatomical regions, reducing the inter-observer variation. These factors led to a statistically significant difference between the error of the model and inter-observer variation in the dental region. In addition, six important indicators in 3D cephalometric analysis were obtained and further proved the clinical feasibility of the proposed model (Table 7).

**Fig. 7:**
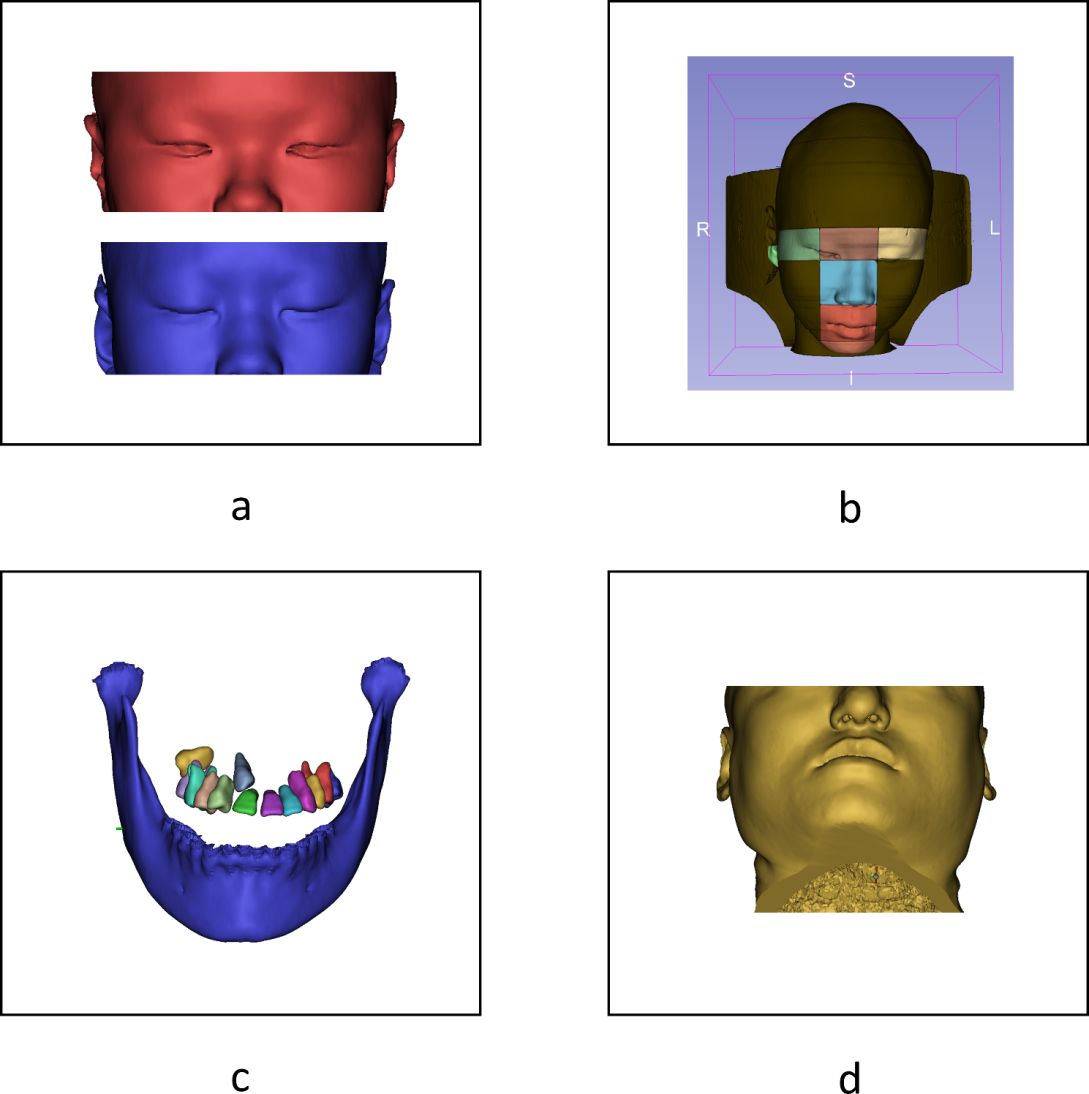
Possible reasons for extremely abnormal results

Despite the desirable results were achieved for most samples, there were still some extremely abnormal errors existed in some cases, which resulted from the following reasons:

- Inconsistency in patients’ eye status: The abnormal detection of landmarks in the periocular soft tissues (sICaL, sICaR, sOCaL, sOCaR) could be caused by differences between the patient’s open and closed eyes (Fig. 7a).
- Inconsistency between the head position of CT data and natural head position [15]: The head positions of some CT images are too forward-leaning, which can interfere with the detection process of the model, especially after region division (Fig. 7b).
- The severity of deformity: Severely abnormal structures impeded the accuracy of landmarks detection. for example, impacted tooth. (Fig. 7c).
- Craniomaxillofacial information missed in CT data: In some cases, the inferior part of chin was not captured, causing the loss of detection for landmarks such as sMe and mMe (Fig. 7d).

Graphic memory footprint refers to the amount of random-access memory (RAM) in graphics card that software references or uses when running. Excessive graphic memory footprint can result in highly expensive training cost and deployment cost, hindering the development of the model and its wide application. The lightweight medical image processing network V-Net is often used as the backbone network for landmarks detection to reduce the graphic memory footprint. The recently proposed 3D UX-Net, characterized by combining the features of the Swin Transformer [16] with convolutional networks, has been increasingly popular for its lightweight architecture and efficient image processing performance. This inspired us to consider replacing V-Net with 3D UX-Net as the backbone of the landmark detection neural network to increase the accuracy of landmarks detection. This study proved that 3D UX-Net outperformed V-Net in 3D landmarks detection.

Due to the high resolution of CT images and limited graphic memory, even lightweight networks such as V-Net or 3D UX-Net was unable to directly process the entire CT images. Thus, the images were often compressed to lower resolutions like 96 × 96 × 96 to reduce the graphic memory footprint. However, image compression will inevitably cause detection inaccuracy. To avoid significant resolution loss, a new region division pattern was designed to divide the entire CT images into 9 regions, allowing landmarks detection to be performed in each region. In this method, the resolution loss was limited to a smaller extent while achieving higher detection accuracy with a lower graphic memory footprint.

Time consumption is one of the most important factors that hinder the implementation of 3D cephalometric analysis in clinical practice. Manual digitization often takes 15 to 25 min for experienced surgeons, and even longer for beginners. Our two-stage deep learning model has effectively solved this problem under lower graphic memory footprint, using only 83s to complete the landmarks digitization task (on NVIDIA RTX 2080Ti 11G).

Comparing to the recent methodologies [6, 7], we proposed a new region division pattern adapt for more and scattered landmarks detection and covered landmarks on all three tissues in CMF CT, including 13 facial soft tissue landmarks, 28 skeletal landmarks and 36 dental landmarks. At the same time, this study has some limitations. There was not sufficient consideration on a method to reduce the occurrence of abnormal results or to ensure the safe use of the model in clinical practice. The abnormal outcomes have also inspired us to further optimize the whole process of automatic landmarks detection, including aligning the head position [17–19], standardizing the process of CT imaging, addressing abnormal data and evaluating the applicability of the model to different patients. Furthermore, in the field of landmarks detection, incorporating the dependency between landmarks into model training is proved to be effective [7, 20]. In this research, the training process were implemented with 9 separate regions from CT images, which inevitably cuts off some potential dependency between landmarks. Restoring the balance between global and local constraints while still maintaining the region division pattern will be further investigated in future.

In summary, the model demonstrated excellent performance in detecting craniomaxillofacial landmarks in CT images while consuming low graphic memory footprint and short time. This model satisfies the clinical requirements for detection accuracy of 3D cephalometric indicators. With further modification and big samples validation, the proposed method could be applied in clinical practice and contributed to the diagnosis and treatment planning of dentomaxillofacial deformities.

### Electronic supplementary material

Below is the link to the electronic supplementary material.

Supplementary Material 1

## Data Availability

The data presented in this study are available on request from Yang Yang (17,732,239,091@163.com). The data are not publicly available due to plans for further research, requirements from the hospital and privacy restrictions.

## Abbreviations

CT: computed tomography
CMF: craniomaxillofacial
TR: teeth region
3D: 3-Dimensional
DL: deep learning
2D: 2-Dimensional
CBCT: cone beam computed tomography
ROI: region of interest
R-ZR: right zygomatic region
L-ZR: left zygomatic region
FR: frontal region
NR: nasal region
MER: mental region
R-MAR: right mandibular region
L-MAR: left mandibular region
SR: sphenoid region
MAD: median absolute deviation
RAM: random-access memory

## References

[1] Cho SM, Kim HG, Yoon SH, Chang KH, Park MS, Park YH, Choi MS (2018). Reappraisal of neonatal Greenstick Skull Fractures caused by birth injuries: comparison of 3-Dimensional reconstructed computed tomography and simple Skull radiographs. World Neurosurg.

[2] Arik SO, Ibragimov B, Xing L (2017). Fully automated quantitative cephalometry using convolutional neural networks. J Med Imaging (Bellingham Wash).

[3] Kunz F, Stellzig-Eisenhauer A, Zeman F, Boldt J (2020). Artificial intelligence in orthodontics evaluation of a fully automated cephalometric analysis using a customized convolutional neural network. J Orofac Orthopedics-Fortschritte Der Kieferorthop.

[4] Lee JH, Yu HJ, Kim MJ, Kim JW, Choi J. Automated cephalometric landmark detection with confidence regions using bayesian convolutional neural networks. BMC Oral Health. 2020;20(1). 10.1186/s12903-020-01256-7 PMC7541217 33028287

[5] Qian J, Luo W, Cheng M, Tao Y, Lin J, Lin H (2020). CephaNN: a multi-head attention network for Cephalometric Landmark Detection. Ieee Access.

[6] Dot G, Schouman T, Chang S, Rafflenbeul F, Kerbrat A, Rouch P, Gajny L (2022). Automatic 3-Dimensional Cephalometric Landmarking via Deep Learning. J Dent Res.

[7] Lang YK, Lian CF, Xiao DQ, Deng HN, Thung KH, Yuan P, Gateno J, Kuang TS, Alfi M, Wang D, Shen L, Xia DG, Yap JJ (2022). Localization of Craniomaxillofacial Landmarks on CBCT images using 3D mask R-CNN and local dependency learning. IEEE Trans Med Imaging.

[8] Cicek O, Abdulkadir A, Lienkamp SS, Brox T, Ronneberger O. 3D U-Net: learning dense volumetric segmentation from sparse annotation. Medical Image Computing and Computer-Assisted Intervention - MICCAI 2016 19th International Conference Proceedings: LNCS 9901. 2016:424 – 32.

[9] Milletari F, Navab N, Ahmadi SA. V-Net: Fully Convolutional Neural Networks for Volumetric Medical Image Segmentation. 4th IEEE International Conference on 3D Vision (3DV); 2016 Oct 25–28; Stanford Univ, Stanford, CA2016.

[10] Liu Q, Deng H, Lian CF, Chen XY, Xiao DQ, Ma L, Chen X, Kuang TS, Gateno J, Yap PT, Xia JJ (2021). SkullEngine: a multi-stage CNN Framework for Collaborative CBCT Image Segmentation and Landmark Detection. Machine learning in medical imaging MLMI. (Workshop).

[11] Lee HH, Bao SX, Huo YK, Landman A. B. 3D UX-Net: A Large Kernel Volumetric ConvNet Modernizing Hierarchical Transformer for Medical Image Segmentation. arXiv. 2022.

[12] Liang CK, Liu SH, Liu Q, Zhang B, Li ZJ (2014). Norms of McNamara’s cephalometric analysis on lateral view of 3D CT imaging in adults from Northeast China. J Hard Tissue Biol.

[13] Cheung LK, Chan YM, Jayaratne YSN, Lo J (2011). Three-dimensional cephalometric norms of chinese adults in Hong Kong with balanced facial profile. Oral Surg Oral Med Oral Pathol Oral Radiol Endodontology.

[14] Ho CT, Denadai R, Lai HC, Lo LJ, Lin HH. Computer-aided planning in orthognathic surgery: a comparative study with the establishment of Burstone Analysis-Derived 3D norms. J Clin Med. 2019;8(12). 10.3390/jcm8122106 PMC6947285 31810228

[15] Tian KY, Li QQ, Wang XX, Liu XJ, Wang X, Li ZL (2015). Reproducibility of natural head position in normal chinese people. Am J Orthod Dentofac Orthop.

[16] Liu Z, Lin YT, Cao Y, Hu H, Wei YX, Zhang Z, Lin S, Guo B. Swin Transformer: Hierarchical Vision Transformer using Shifted Windows. 18th IEEE/CVF International Conference on Computer Vision (ICCV); 2021 Oct 11–17; Electr Network2021.

[17] Damstra J, Fourie Z, Ren YJ (2010). Simple technique to achieve a natural position of the head for cone beam computed tomography. Br J Oral Maxillofacial Surg.

[18] Kim DS, Yang HJ, Huh KH, Lee SS, Heo MS, Choi SC, Hwang SJ, Yi WJ (2014). Three-dimensional natural head position reproduction using a single facial photograph based on the POSIT method. J Cranio-Maxillofacial Surg.

[19] Schatz EC, Xia JJ, Gateno J, English JD, Teichgraeber JF, Garrett FA (2010). Development of a technique for Recording and transferring natural head position in 3 dimensions. J Craniofac Surg.

[20] Payer C, Stern D, Bischof H, Urschler M (2019). Integrating spatial configuration into heatmap regression based CNNs for landmark localization. Med Image Anal.

