# Supplementary material for: Automatic craniomaxillofacial landmarks detection in CT images of individuals with dentomaxillofacial deformities by a two-stage deep learning model
Source: BMC Oral Health. 2023 Nov 17;23:876. doi: 10.1186/s12903-023-03446-5 (PMC10657133; doi:10.1186/s12903-023-03446-5)
Supplement: Supplementary file 1 — Supplementary Material 1 [file 12903_2023_3446_MOESM1_ESM.pdf]

Supplementary Table 1

Landmarks	Definition
**t11**	The midpoint of incisal ridge of right maxillary central incisor.
**t11A**	The apex of right maxillary central incisor.
**t12**	The midpoint of incisal ridge of right maxillary lateral incisor.
**t12A**	The apex of right maxillary central incisor.
**t13**	The cusp of right maxillary canine.
**t13A**	The apex of right maxillary canine.
**t14**	The buccal cusp of right maxillary first premolar.
**t16**	The mesiobuccal cusp of right maxillary first molar.
**t17**	The mesiobuccal cusp of right maxillary second molar.
**t21**	The midpoint of incisal ridge of left maxillary central incisor.
**t21A**	The apex of left maxillary central incisor.
**t22**	The midpoint of incisal ridge of left maxillary lateral incisor.
**t22A**	The apex of left maxillary central incisor.
**t23**	The cusp of left maxillary canine.
**t23A**	The apex of left maxillary canine.
**t24**	The buccal cusp of left maxillary first premolar.
**t26**	The mesiobuccal cusp of left maxillary first molar.
**t27**	The mesiobuccal cusp of left maxillary second molar.
**t31**	The midpoint of incisal ridge of left mandibular central incisor.
**t31A**	The apex of left mandibular central incisor.
**t32**	The midpoint of incisal ridge of left mandibular lateral incisor.
**t32A**	The apex of left mandibular central incisor.
**t33**	The cusp of left mandibular canine.
**t33A**	The apex of left mandibular canine.
**t34**	The buccal cusp of left mandibular first premolar.
**t36**	The mesiobuccal cusp of left mandibular first molar.
**t37**	The mesiobuccal cusp of left mandibular second molar.
**t41**	The midpoint of incisal ridge of right mandibular central incisor.
**t41A**	The apex of right mandibular central incisor.
**t42**	The midpoint of incisal ridge of right mandibular lateral incisor.
**t42A**	The apex of right mandibular central incisor.
**t43**	The cusp of right mandibular canine.
**t43A**	The apex of right mandibular canine.
**t44**	The buccal cusp of right mandibular first premolar.
**t46**	The mesiobuccal cusp of right mandibular first molar.
**t47**	The mesiobuccal cusp of right mandibular second molar.
**sN**	The nasion on soft tissue.
**sICa**	The internal canthal point of the palpebral fissure.
**sOCa**	The external canthal point of the palpebral fissure.
**sCh**	The point at mouth corner.
**sChU**	The most prominent midpoint of the upper vermilion line.
**sChB**	The most prominent midpoint of the upper vermilion line.
**sPog**	The most anterior midpoint of the chin on the outline of the soft tissue.
**sPrn**	The most anterior point at the nasal tip.
**sMe**	The most inferior midpoint of the chin on the outline of the soft tissue.
**uA**	The most posterior midline point on the premaxilla between the anterior nasal spine and prosthion.
**uANS**	The sharp anterosuperior projection at the anterior extremity of the line of union of the two maxillae.
**uPNS**	The most posterior midpoint of the posterior nasal spine of the palatine bone.
**uPo**	The most lateral point on the roof of the bony external acousticmeatus, vertically over the middle of the meatus.
**uOr**	The most inferior point of infraorbital margin.

**uN**	The point on the skull corresponding to the middle of the nasofrontal suture.
**uBa**	The most anterior point at the base of foramen magnum.
**uS**	The center of the sella turcica on the midsagittal plane.
**uZy**	The most lateral point of zygomatic arch.
**uZF**	The most medial point of fronto-zygomatic suture.
**u0**	The most lateral point of the piriform aperture.
**u1**	The midpoint of zygomatic alveolar ridge.
**mCo**	The most superior point on the head of the mandible
**mGo**	Lines to the posterior margin of the mandibular vertical ramus and inferior margin of the mandibular body or horizontal ramus.
**mB**	The most posterior midline point, above the chin and on the mandible between the infradentale and the pogonion.
**mPog**	The most anterior p(1)oint of the mandible in the midline.
**mGn**	The midpoint of mPog and mMe on the mandible in the midline.
**mMe**	The most inferior point of the mandible in the midline.
**mGF**	The most anterior point of mental foramen.

Supplementary Figure 1

Annotation for Stage 1 of the model

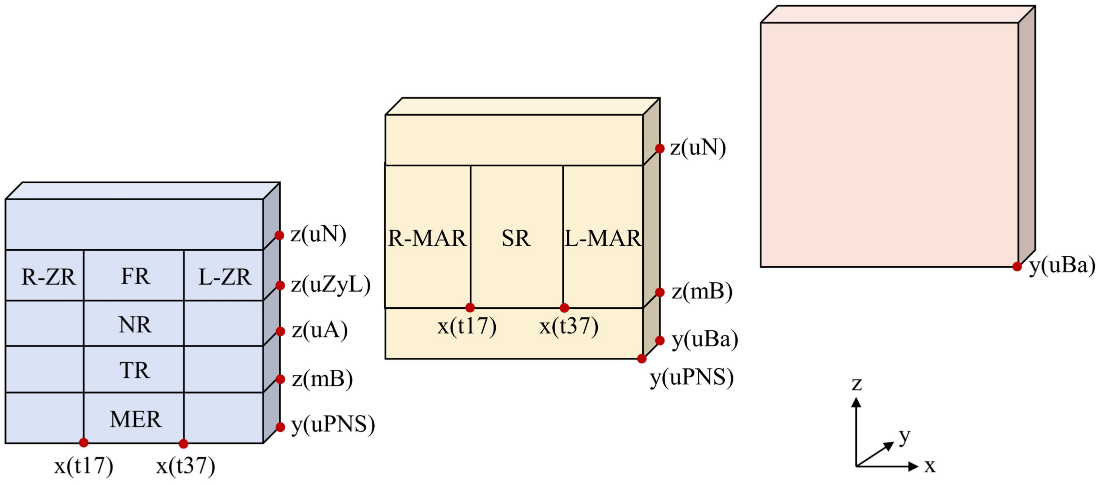

Supplementary Figure 2

A block diagram of UX-Net showing different layers

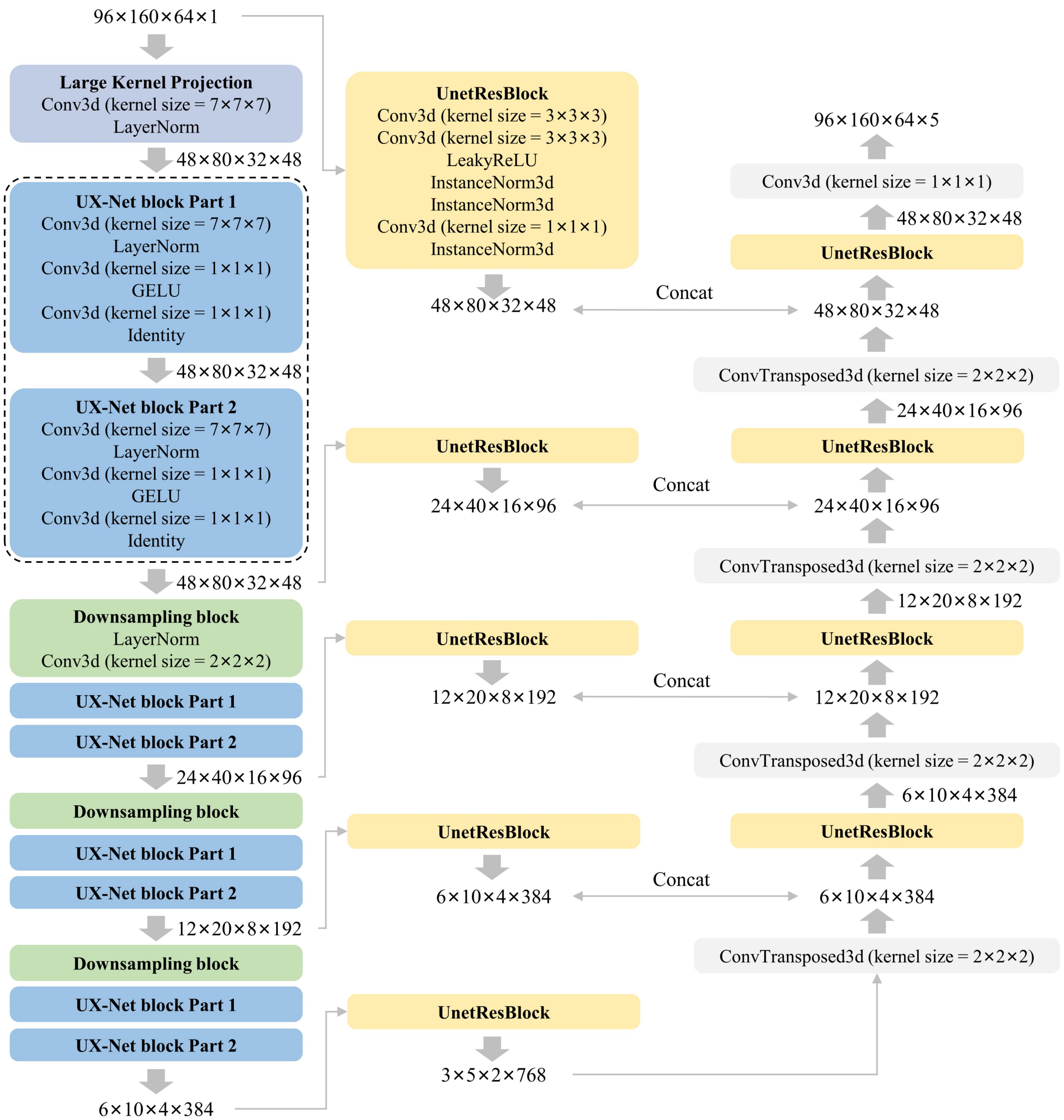

Supplementary Table 2

Input/output size of data and the landmarks in nine regions

Region	Input size	Output size	Number of Landmarks	Detail
**R-ZR**	160 × 160 × 64 × 1	160 × 160 × 64 × 5	4	sOCaR, uOrR, uZFR, uZyR
**L-ZR**	160 × 160 × 64 × 1	160 × 160 × 64 × 5	4	sOCaL, uOrL, uZFL, uZyL
**FR**	96 × 160 × 64 × 1	96 × 160 × 64 × 5	4	uN, sN, sICaR, sICaL
**NR**	96 × 160 × 96 × 1	96 × 160 × 96 × 9	8	u0R, u0L, uA, uANS, sSN, sPrn, u1R, u1L
**TR**	96 × 160 × 96 × 1	96 × 160 × 96 × 41	40	sChU, sChB, sChL, sChR, t11, t11A, t12, t12A, t13, t13A, t14, t15, t16, t17, t21, t21A, t22, t22A, t23, t23A, t24, t25, t26, t27, t31, t31A, t32, t32A, t33, t33A, t34, t35, t36, t37, t41, t41A, t42, t42A, t43, t43A, t44, t45, t46, t47
**MER**	96 × 160 × 64 × 1	96 × 160 × 64 × 9	8	sPog, sMe, mB, mPog, mGn, mMe, mGFR, mGFL
**R-MAR**	160 × 64 × 160 × 1	160 × 64 × 160 × 4	3	uPoR, mCoR, mGoR
**L-MAR**	160 × 64 × 160 × 1	160 × 64 × 160 × 4	3	uPoL, mCoL, mGoL
**SR**	96 × 96 × 160 × 1	96 × 96 × 160 × 4	3	uBa, uS, uPNS
